# A new mosaic pattern in glioma vascularization: exogenous endothelial progenitor cells integrating into the vessels containing tumor-derived endothelial cells

**DOI:** 10.18632/oncotarget.1885

**Published:** 2014-04-20

**Authors:** Xiao Chen, Jingqin Fang, Shunan Wang, Heng Liu, Xuesong Du, Jinhua Chen, Xue Li, Yizeng Yang, Bo Zhang, Weiguo Zhang

**Affiliations:** ^1^ Department of Radiology, Institute of Surgery Research, Daping Hospital, Third Military Medical University, Chongqing, 400042, China; ^2^ Division of Gastroenterology, Department of Medicine, University of Pennsylvania Perelman School of Medicine, Philadelphia, PA, USA; ^3^ Four and the State key laboratory of Trauma, Burns and Combined Injury, Institute of Surgery Research, Daping Hospital, Third Military Medical University, Chongqing, 400042, China; ^4^ State key laboratory of Trauma, Burns and Combined Injury, Institute of Surgery Research, Daping Hospital, Third Military Medical University, Chongqing, 400042, China

**Keywords:** endothelial progenitor cells, C6 glioma cells, glioma, angiogenesis, magnetic resonance imaging

## Abstract

Emerging evidence suggests that glioma stem-like cells (GSCs) transdifferentiating into vascular endothelial cells (ECs) possibly contributes to tumor resistance to antiangiogenic therapy. Endothelial progenitor cells (EPCs), showing active migration and incorporation into neovasculature of glioma, may be a good vehicle for delivering genes to target GSCs transdifferentiation. Here, we found a new mosaic pattern that exogenous EPCs integrated into the vessels containing the tumor-derived ECs in C6 glioma rat model. Further, we evaluated the effect of these homing EPCs on C6 glioma cells transdifferentiation. The transdifferentiation frequency of C6 glioma cells and the expressions of key factors on GSCs transdifferentiation, i.e. HIF-1α, Notch1, and Flk1 in gliomas with or without EPCs transplantation showed no significant difference. Additionally, magnetic resonance imaging could track the migration and incorporation of EPCs into glioma *in vivo*, which was confirmed by Prussian blue staining. The number of magnetically labeled EPCs estimated from T_2_ maps correlated well with direct measurements of labeled cell counts by flow cytometry. Taken together, our findings may provide a rational base for the future application of EPCs as a therapeutic and imaging probe to overcome antiangiogenic resistance for glioma and monitor the efficacy of this treatment.

## INTRODUCTION

Glioma, which is the most common primary brain tumor in central nervous system in adults, is one of the most vascular-rich tumors and has short median survival, especially glioblastoma multiforme [1]. Anti-vascular endothelial growth factor (VEGF) therapy has had significant efficacy in glioma with more than half of responders, but this effect is transient in most patients, acquired antiangiogenic resistance may occur [2, 3].

Emerging evidence has detailed several mechanisms, including enhanced invasion [4 - 6] and alternative angiogenesis [7-10], by which glioma adapts to and circumvents antiangiogenic therapy. Recent studies demonstrate that a population of glioma stem-like cells (GSCs) transdifferentiate into vascular endothelial cells (ECs) within glioma, possibly via an intermediate endothelial progenitor cells (EPCs), which may provide new perspectives on the mechanisms of the resistance to anti-VEGF therapy currently in use [9, 10]. Therefore, identifying new therapeutic strategies for antiangiogenic therapy in glioma is a high priority.

Owing to their unique property of migration to pathological lesions, stem cells are considered to be used as a delivery vehicle for therapeutic genes to tumors, especially for glioma [11 -13]. EPCs, a subpopulation of pluripotent hematopoietic stem cells (HSCs), could migrate actively to glioma [14-16], being incorporated directly in the neovasculature with high specificity [17-19]. However, whether EPCs integrate into the vessels which contain the tumor-derived ECs or not remains unknown. If that is the case, EPCs may be a best vehicle to deliver the therapeutic genes, targeting GSCs transdifferentiation more sufficiently and effectively.

In the present study, we found a new mosaic pattern in glioma vascularization, i.e. exogenous EPCs integrated into the vessels containing the tumor-derived ECs in C6 glioma rat model and these homing EPCs exerted no promoting effect on the transdifferentiation of C6 glioma cells into vascular ECs. Our work may provide useful support for the future application of EPCs as a therapeutic vehicle to overcome anti-angiogenic resistance for glioma.

## Results

### Transdifferentiation of C6 glioma cells into endothelial cells *in vivo* and *in vitro*

**Figure 1:**
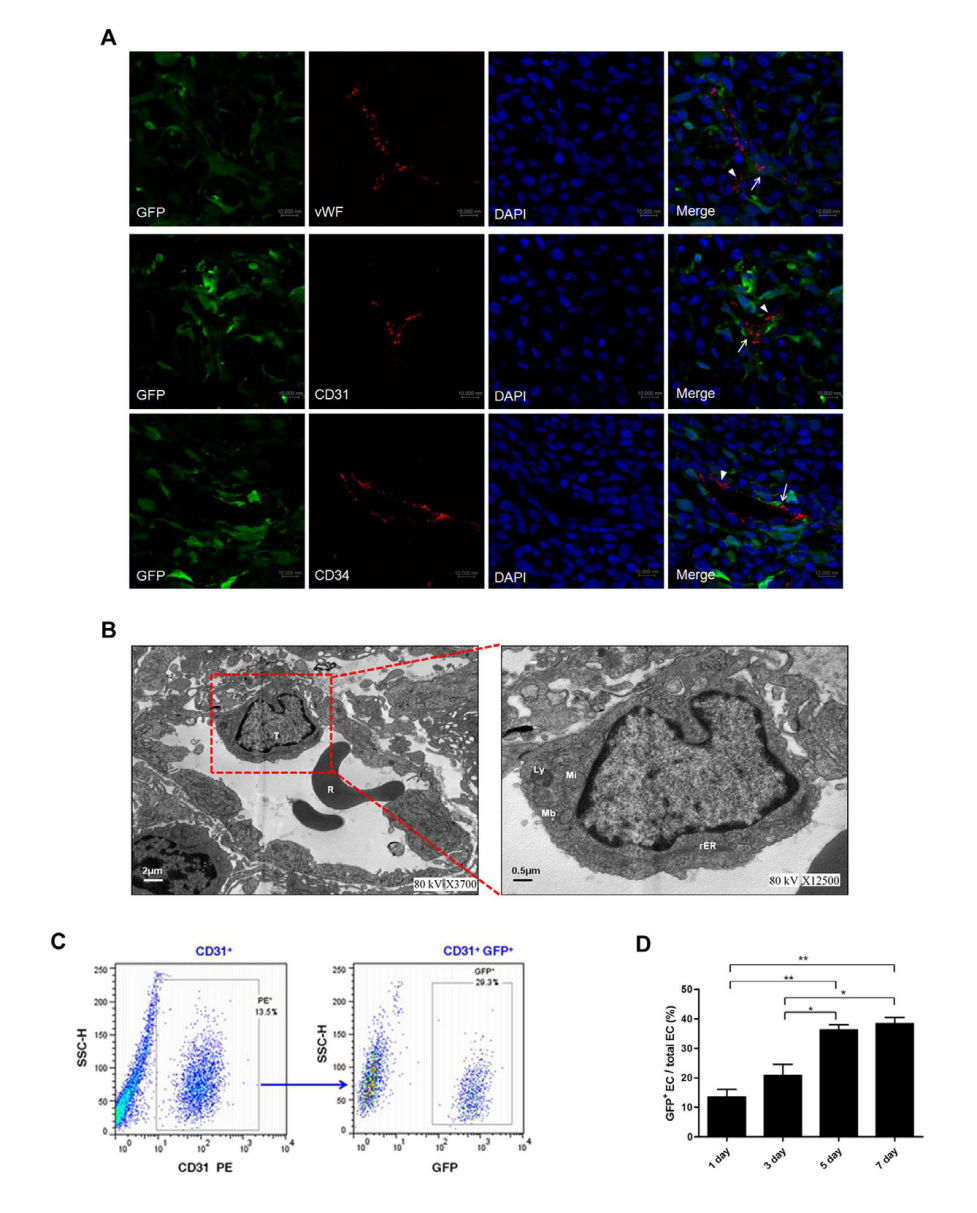
Transdifferentiation of C6 glioma cells into endothelial cells (ECs) *in vivo* A. Representative images of tumor-derived ECs and regular ECs lined the vessel lumen in gliomas of the group with DPBS transplantation (control group). Regular ECs (arrowheads) expressed EC markers vWF, CD31, and CD34 (red), but not the tumor marker GFP. In contrast, tumor-derived ECs (arrows) expressed both GFP (green) and EC markers vWF, CD31, and CD34. Scale bar: 37.5 μm. B. Representative ultrastructural view of the vessel containing tumor-derived ECs. The right panel is the amplification of the frame in left panel, which shows that transdifferentiated C6 glioma cells had similar ultrastructural characteristics as ECs with many lysosomes, multivesicular bodies, plenty of mitochondria of small size with less cristae and a few rER in cytoplasms. T, tumor-derived ECs; R, erythrocyte; Mi, mitochondria; Ly, lysosome; rER, rough endoplasmic reticulum; Mb, multivesicular body. Scale bars are indicated. C. Representative result of flow cytometry for dissociated glioma tissue in control group. ECs were CD31^+^ and constituted 13.5% of the whole tumor (left), and GFP^+^ ECs (tumor-derived ECs) represented 29.3% of total ECs (right). D. Quantification of transdifferentiation frequency of C6 glioma cells in glioma tissue on day 1, 3, 5, 7 after DPBS transplantation in control group. Data are mean ± SD from three independent experiments. *, p<0.05; **, p<0.01.

To investigate the tumor vasculature, we carried out immunofluorescence by confocal microscopy using the endothelial antigens vWF, CD31, and CD34 as markers. As shown in Fig. 1A, a regular EC, where the GFP in tumor cells is completely distinct from vWF, only expressed the endothelial antigens. However, we also found that some ECs expressed not only endothelial antigens but also GFP, which suggests these ECs may be originated from tumor cells. Transmission electron microscopy analysis also unveiled that C6 glioma cells integrated into functional tumor vessels and shared the ultrastructural characteristics of ECs (Fig. 1B) that is in agreement with Zhao et al. [20]. To confirm the presence of GFP^+^ ECs, we examined dissociated tumors by flow cytometry. Similar to the results of confocal microscopy and transmission electron microscopy, 12.7% - 43.2% of ECs were positive for GFP (average of 31.20 ± 11.96%) (Fig. 1C). Additionally, the transdifferentiation frequency of C6 glioma cells was increased in a time-dependent manner, i.e. depending on the size of the tumor (Fig. 1D).

**Figure 2:**
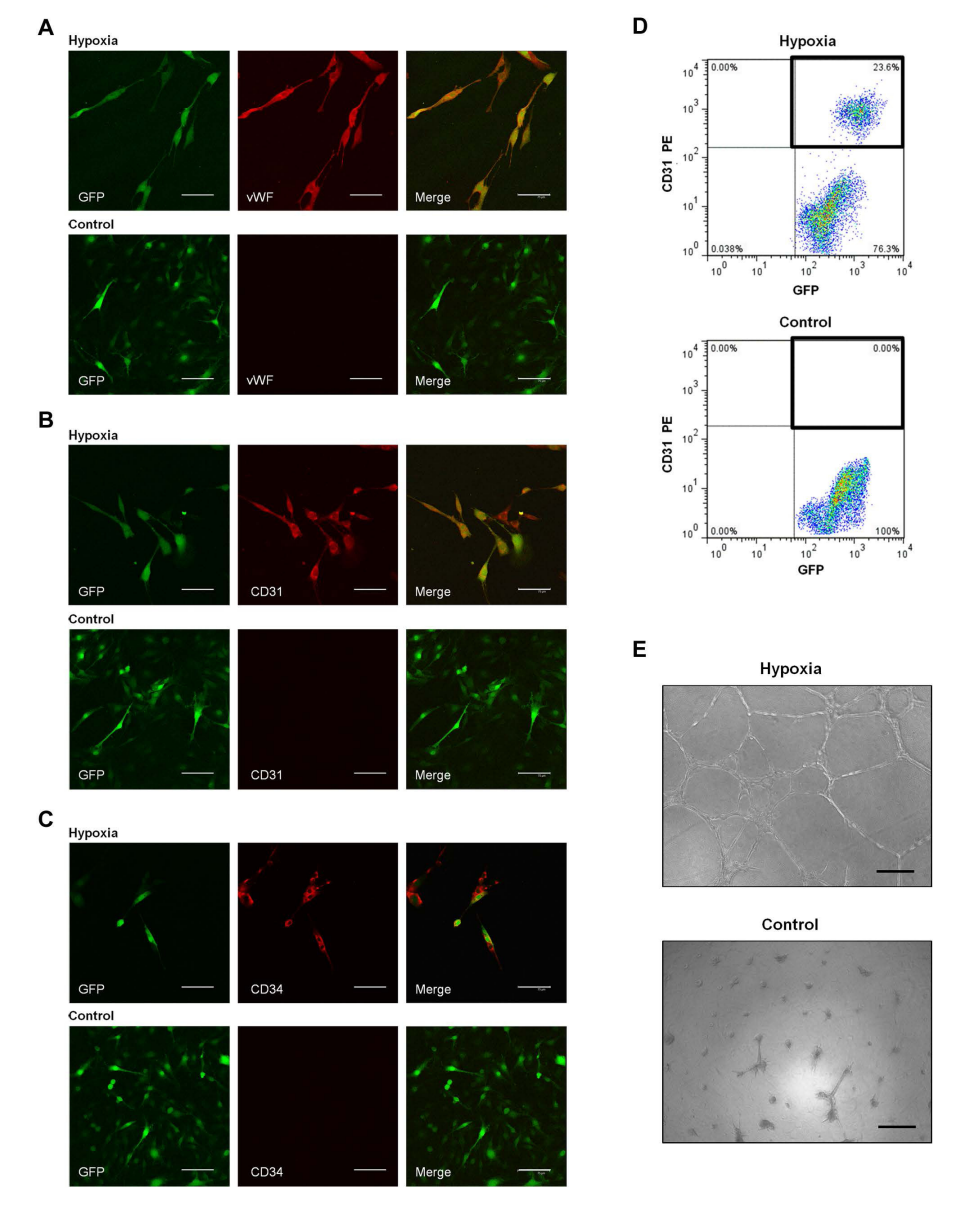
Transdifferentiation of C6 glioma cells into ECs *in vitro* A-C. Representative images of C6 glioma cells in hypoxia group (cultured in endothelial differentiation medium in hypoxia; *top*) and control group (cultured in DMEM/F_12_ in normoxia; *bottom*) *in vitro*. A, for vWF (red); B, for CD31 (red); C, for CD34 (red). Scale bar: 75 μm. D. Flow cytometry of CD31 and GFP expression in C6 glioma cells in hypoxia group (top) and control group (bottom). E. Tube formation assay of C6 glioma cells in hypoxia group (top) and control group (bottom). Scale bar: 100 μm.

We next attempted to induce C6 glioma cells to differentiate into ECs *in vitro*. C6 glioma cells were cultured in endothelial differentiation medium in hypoxia. Immunofluorescence analysis showed the co-expression of GFP and endothelial antigens vWF, CD31 and CD34 (Fig. 2A-C). Flow cytometry analysis also revealed that a substantial proportion of C6 glioma cells about 36.8% co-expressed CD31 and GFP, representative images had been shown in Fig. 2D. To investigate the vasculogenic capacity of C6 glioma cells, we performed tube formation assay that commonly recapitulates the ability of endothelial cells to develop vasculature *in vitro*. When grown on Matrigel, C6 glioma cells formed vascular networks reminiscent of normal endothelium (Fig. 2E).

### Role of HIF-1α, Notch 1 and Flk1 in C6 glioma cells transdifferentiation

**Figure 3:**
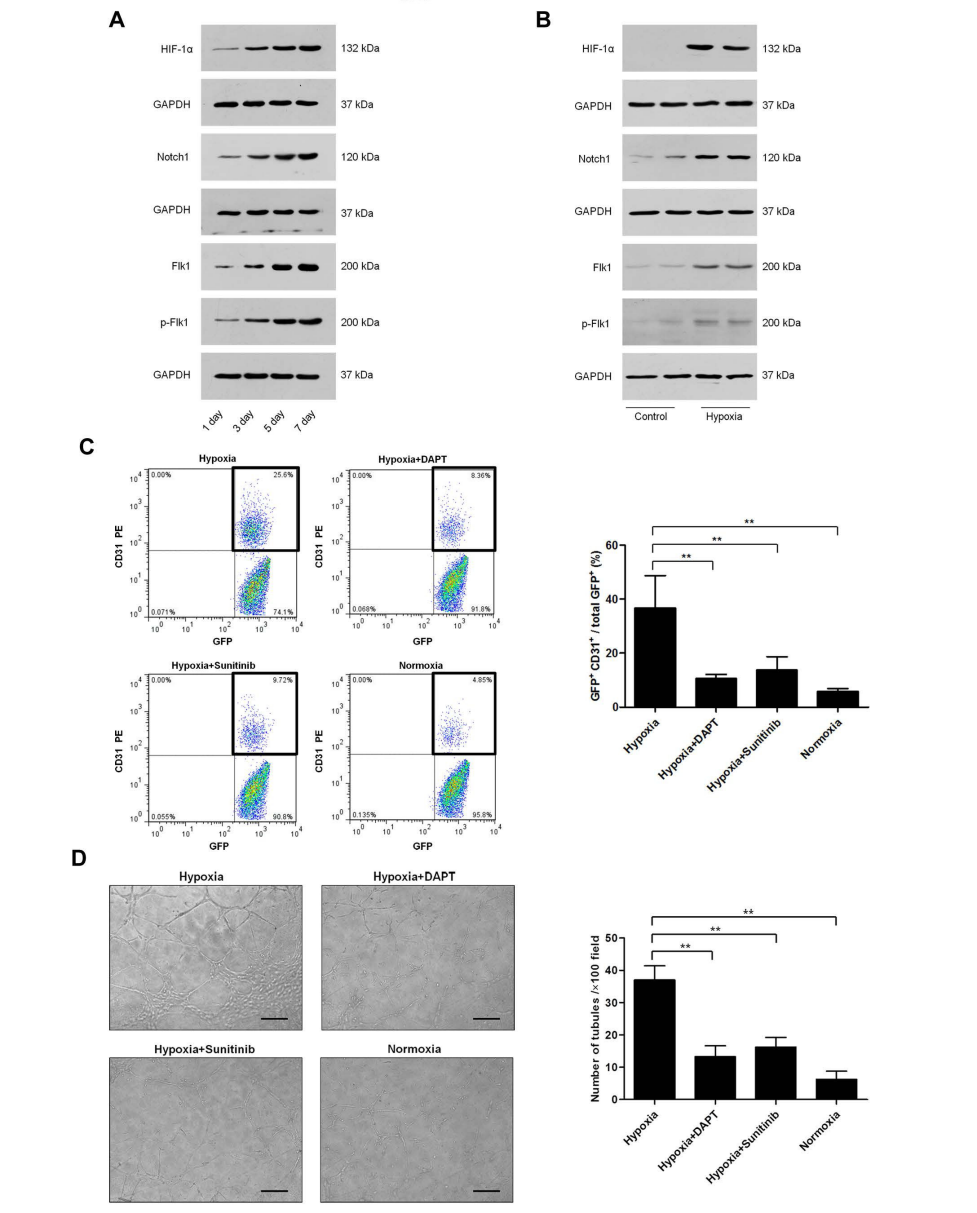
Role of HIF-1α, Notch1 and Flk1 in C6 glioma cells transdifferentiation A-B. Western blot analysis of HIF-1α, Notch1, Flk1, and p-Flk1 in gliomas of control group (A) and transdifferentiation-induced C6 glioma cells lysates (B). GAPDH blot serves as loading control. C. Flow cytometry of CD31 and GFP expression in C6 glioma cells cultured for 24 h in various conditions, including endothelial differentiation medium in hypoxia, endothelial differentiation medium containing DAPT (5 μM) or sunitinib (5 μM) in hypoxia, and endothelial differentiation medium in normoxia. Analysis of transdifferentiation frequency of C6 glioma cells in different groups. D. Tube formation assay of C6 glioma cells cultured in various conditions which were indicated before. The number of tubules in different groups were counted and analysed. Data are mean ± SD from three independent experiments. **, p<0.01.

Recent studies demonstrate that HIF-1α [10], Notch [9, 21] and VEGFR2 [9, 22] signaling may mediate distinct steps in the transdifferentiation of GSCs into ECs. To further elucidate the underlying mechanism of C6 glioma cells transdifferentiating into ECs, we evaluated the protein expression of HIF-1α, Notch1, Flk1, and p-Flk1 in glioma tissue and C6 glioma cells by Western blot. As we showed before, the transdifferentiation frequency of C6 glioma cells was higher in large tumors than in small tumors (Fig. 1D). Fig. 3A showed that HIF-1α, Notch1, Flk1, and p-Flk1 expressions were increased in the glioma tissue in a time-dependent manner, i.e. depending on the size of the tumor. *In vitro*, the expressions of HIF-1α, Notch1, Flk1, and p-Flk1 in transdifferentiation-induced C6 glioma cells were significantly higher than the control (Fig. 3B).

To further explore the enhanced expression of these factors, transdifferentiation-induced C6 glioma cells were treated with DAPT (γ-secretase inhibitor that effectively inhibits Notch signaling) [21], sunitinib (VEGFR2 tyrosine kinase inhibitor currently in clinical trial) [23], normoxia or vehicle control for 24 h, respectively. Followed by, we detected the transdifferentiation frequency by flow cytometry and performed tube formation assay. Compared with the hypoxia group, the transdifferentiation frequency of treated cells was reduced about 71%, 62% and 84%, respectively (Fig. 3C), and the ability of tube formation was inhibited about 64%, 55% and 83%, respectively (Fig. 3D). Therefore, these results suggest that HIF-1α, Notch1, and Flk1 may be important factors for C6 glioma cells transdifferentiation.

### *In vivo* MRI tracking and quantifying of EPCs incorporation into tumor

**Figure 4:**
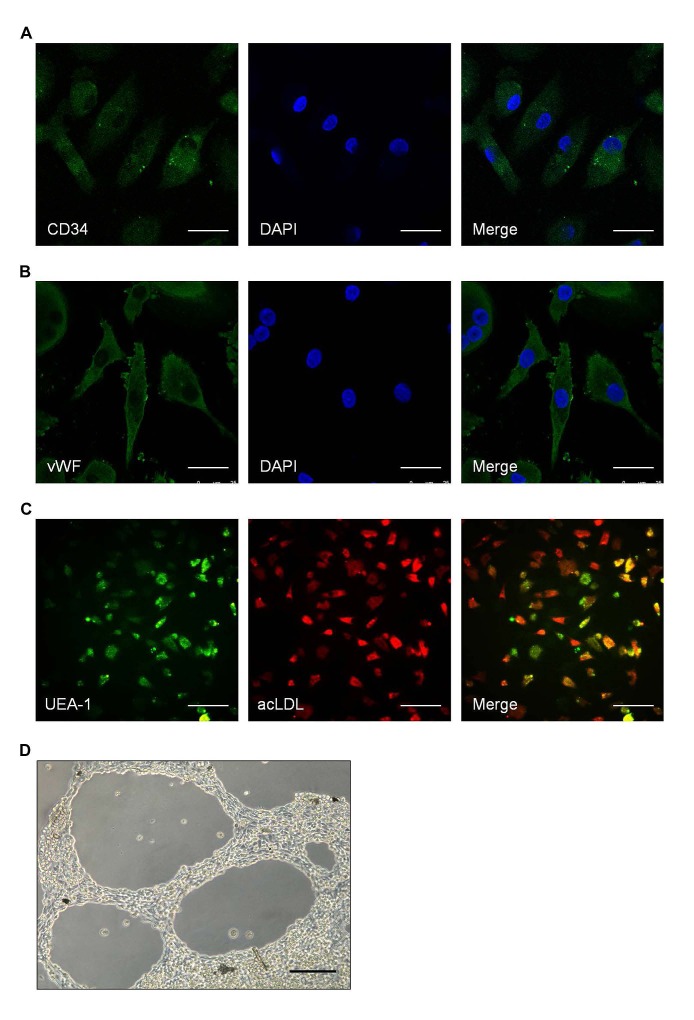
Identifying the characteristics of spleen-derived endothelial progenitor cells (EPCs) A-B. Representative images of the surface markers on spleen-derived EPCs. A for CD34 (green); B for vWF (green). Scale bar: 25 μm. C. Representative images of EPCs uptake of DiI-acLDL and binding of FITC-UEA-1. Scale bar: 100 μm. D. Representative images of EPCs angiogenesis *in vitro*. Scale bar: 100 μm

To identify the characteristics of EPCs, immunocytochemistry and *in vitro* angiogenesis assay were performed to detect the surface markers and function of EPCs. EPCs were found to express high amount of CD34 (Fig. 4A) and vWF(Fig. 4B). Most adherent cells showed uptake of DiI-acLDL and binding of FITC-UEA-1 (Fig. 4C). *In vitro* angiogenesis assay showed the ability of EPCs to form neovasculature (Fig. 4D).

**Figure 5:**
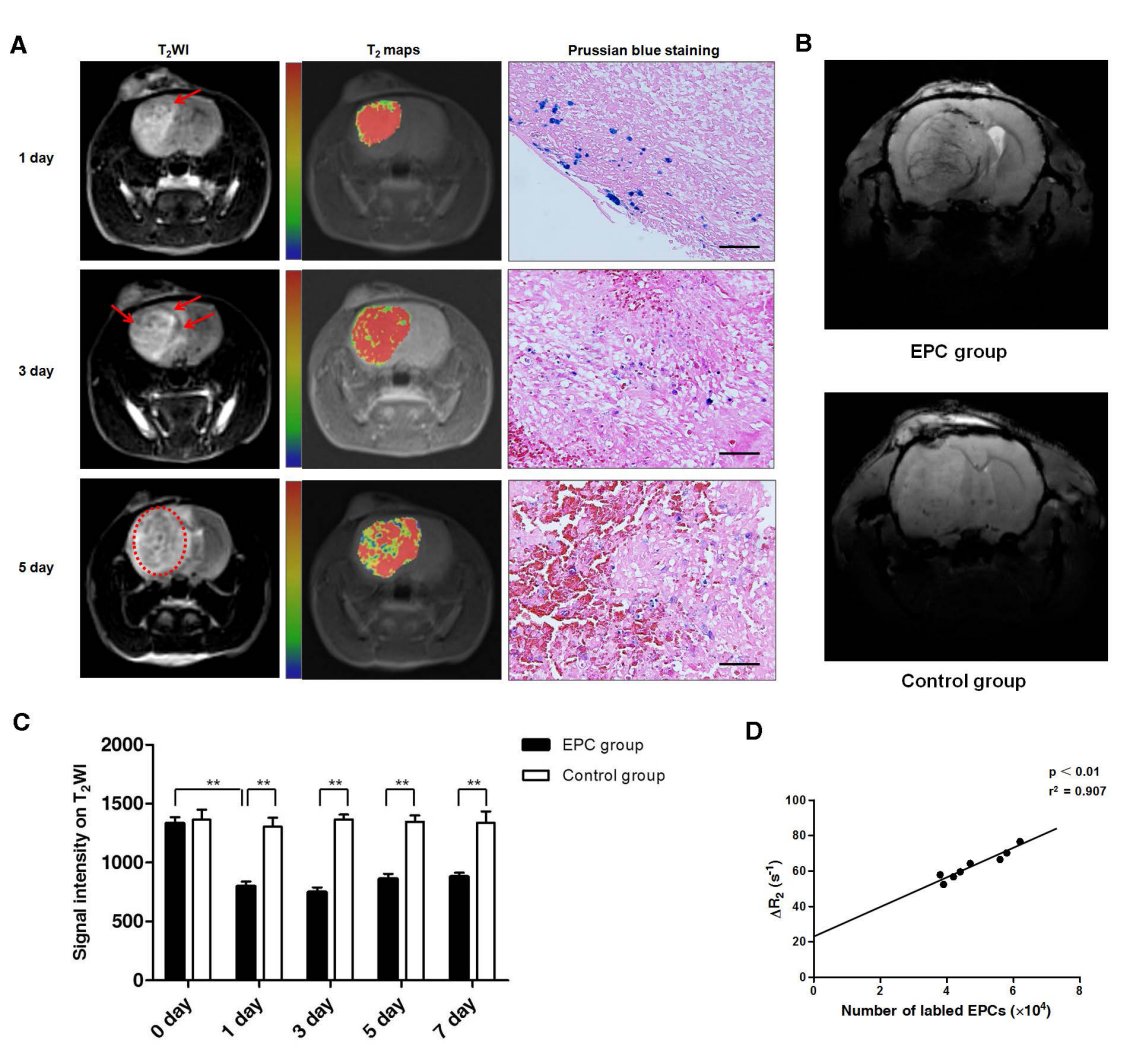
Magnetic resonance imaging (MRI) tracking and quantifying of EPCs incorporation into tumor *in vivo* A. Representative T_2_-weighted imaging (T_2_WI) and T_2_ maps images of rats with glioma on 1, 3, 5 days after EPCs transplantation from EPC group using 3.0 T MRI. Glioma was the region with hyperintensity on T_2_WI images. Point-like or linear hypointensity (dark area; arrows or dotted ring) was found in glioma and the dark area enlarged as time went on. The same signal changes were found in T_2_ maps. In T_2_ maps, red and blue represent relative higher- and lower-value, respectively. Histology with Prussian blue staining showed iron-positive cells (blue particles) at the corresponding site of areas of point-like or linear hypointensity on MRI. Scale bar: 100 μm. B. Representative susceptibility-weighted imaging (SWI) of rats with glioma on 3 days after transplantation in EPC group and control group using 7.0 T MRI. Hypointensity was along/around the vessels in EPC group, while no such changes were found in control group. C. Changes of signal intensity on T_2_ maps in EPC group and control group were analysed. Data are mean ± SD from three independent experiments. **, p<0.01. D. Correlation between number of labeled EPCs detected by flow cytometry and MRI △R2 values. Regression line: y=23.359+8.214x, r^2^=0.907, p<0.01.

To track the migration of intravenously injected, magnetically labeled EPCs into glioma *in vivo*, all 48 rats were scanned using 3.0 T MRI before and after EPCs transplantation. The tumors on T_2_WI were patch-like areas of hyperintensity, with no hypointensity inside before EPCs transplantation. On day 1 after EPCs transplantation, hypointensity could be detected on T_2_WI mostly at the rim of the tumor. Three days after transplantation, hypointensity became more significant, and the hypointense areas were enlarged. Five or seven days after transplantation, the hypointense area nearly covered the entire tumor foci (Fig. 5A). However, no such significant signal changes were observed in the control group. There was a significant difference between EPC group and control group in signal intensity on the T_2_WI at each time point after transplantation (Fig. 5C). Consistent with the MRI findings, Prussian blue staining showed that the distribution of Prussian blue-positive cells were from the edge to the center of the tumor over time (Fig. 5A). While, no Prussian blue-positive cells were found in the control group. To further study of the magnetically labeled EPCs integrating into the vessels in glioma, 7.0 T MRI was performed before and after EPCs transplantation. SWI, a highly sensitive way of identifying iron storage [24], can be used to depict both cerebral veins and arteries [25]. In EPCs transplanted group, the hypointensity was obvious along the vessels on SWI. While, no such significant signal changes were observed in the control group (Fig. 5B).

As is reported, MRI sequences such as T_2_ maps can be used to quantify the iron concentration in tissue, making quantification of therapeutic agent delivery easier [26-28]. Here, we noted the linear correlation between the number of labeled cells detected by flow cytometry and corresponding △R2 value (r^2^=0.907, p<0.01; Fig. 5D). Taken together, these results suggest that MRI can be used to quantitatively monitor the delivery of labeled EPCs *in vivo*.

### Exogenous EPCs integrating into the vessels containing the tumor-derived ECs

**Figure 6:**
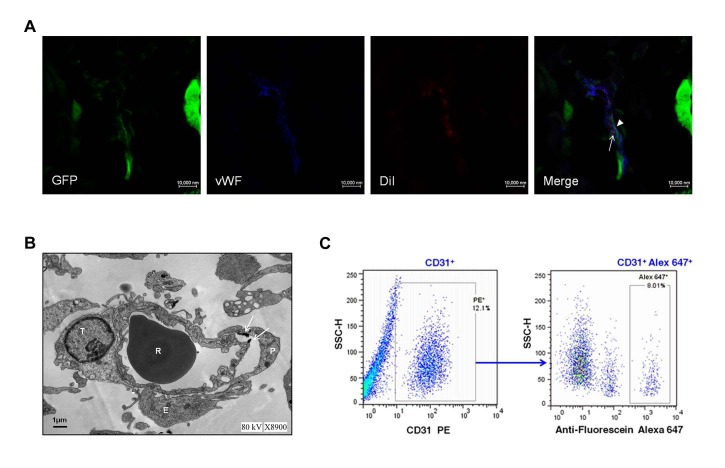
Exogenous endothelial progenitor cells (EPCs) integrating into the vessels containing the tumor-derived ECs A. Representative images of exogenous EPCs, tumor-derived ECs and regular ECs lined the vessel lumen in gliomas of EPC group. EPCs (arrows) were labeled with DiI (red) but also expressed EC marker CD31 (blue). In contrast, tumor derived-ECs (arrowheads) expressed both the GFP (green) and CD31, and regular ECs only expressed CD31. Scale bar: 10 μm. B. Representative ultrastructural view of regular ECs, tumor-derived ECs, and USPIO-labeled EPCs composed a vessel. T, tumor-derived EC; P, EPC; E, EC; R, erythrocyte. Arrows depict the magnetic nanoparticle USPIO. Scale bar: 1μm. C. Representative result of flow cytometry for dissociated glioma tissue in EPC group. ECs were CD31^+^ and constituted 12.1% of the whole tumor (left), and CD31^+^ Alex 647^+^ (exogenous EPCs) represented 8.01% of total ECs (right).

To test our hypothesis that EPCs may integrate into the vessels containing the tumor-derived ECs, we investigated this neovascularization in glioma tissue by immunofluorescence and transmission electron microscopy. Interestingly, we found that regular ECs expressing only endothelial antigens, cells expressing both endothelial antigens and GFP (tumor-derived ECs), and cells expressing both endothelial antigens and DiI (EPCs) existed in one vessel by confocal microscopy (Fig. 6A). Transmission electron microscopy analysis also showed that regular ECs, tumor-derived ECs, and USPIO-labeled EPCs composed a vessel (Fig. 6B), further supporting the integration of EPCs into the vessels containing the tumor-derived ECs. In addition, flow cytometry was performed to detect the presence of EPCs integration into the tumor vessels. About 8.06% of total ECs in tumor were positive for Alexa Fluor 647 (Fig. 6C), some of which integrated into the vessels containing the tumor-derived ECs.

### Effects of exogenous EPCs on C6 glioma cells transdifferentiation

**Figure 7:**
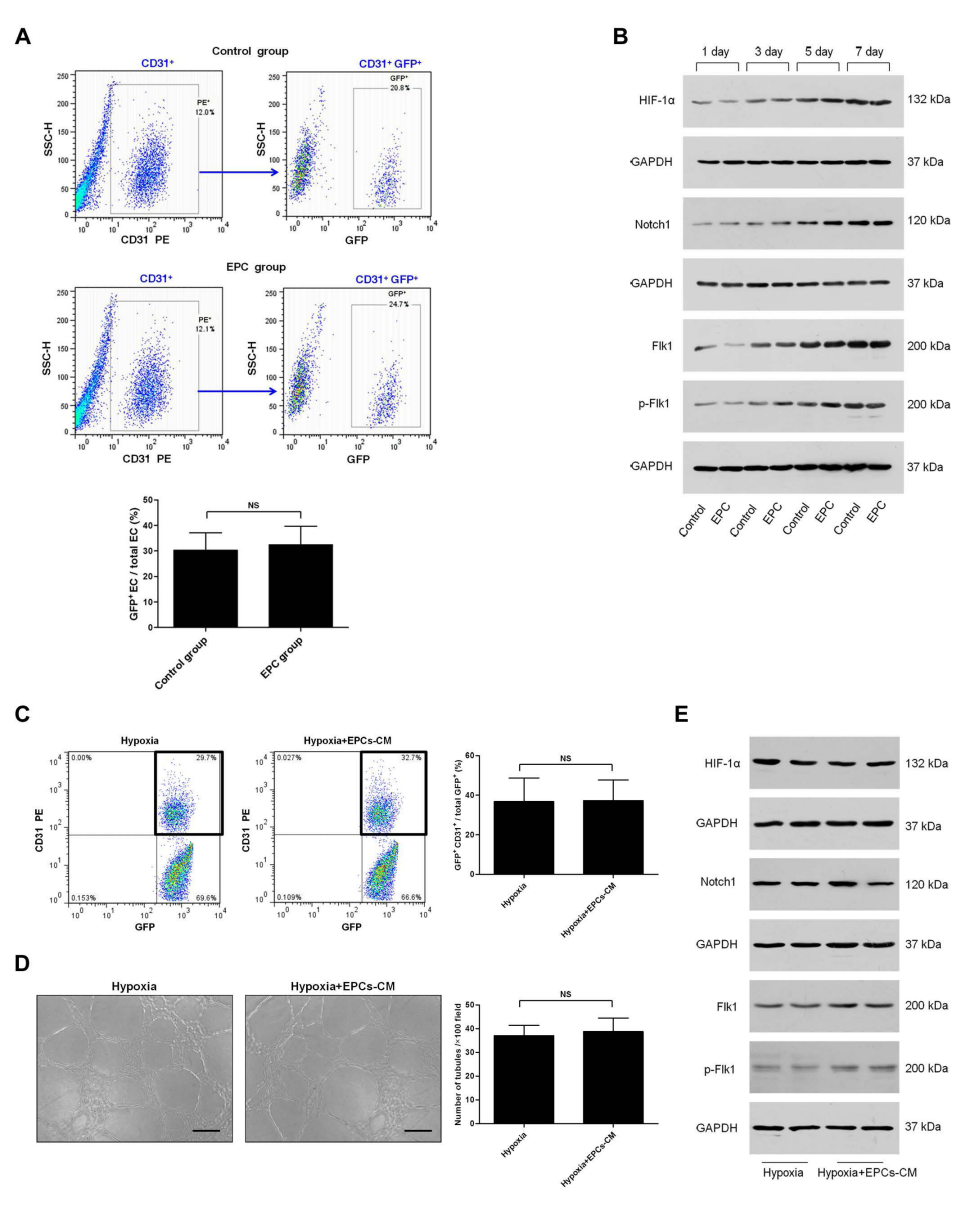
Effects of exogenous EPCs on C6 glioma cells transdifferentiation A. Representative result of flow cytometry for dissociated glioma tissue in control group (top) and EPC group (middle). Analysis of transdifferentiation frequency of C6 glioma cells in gliomas of these two groups (bottom). B. Western blot analysis of HIF-1α, Notch1, Flk1, and p-Flk1 in gliomas of control group and EPC group on 1, 3, 5, 7 days after DPBS or EPCs transplantation. GAPDH blot serves as loading control. C. Flow cytometry of CD31 and GFP expression in C6 glioma cells cultured for 24 h in two conditions, including endothelial differentiation medium (left) or endothelial differentiation medium containing 50% (v/v) EPCs conditioned medium (EPCs-CM; middle) in hypoxia. Analysis of transdifferentiation frequency of C6 glioma cells in these two groups (right). D. Tube formation assay of C6 glioma cells cultured in these two conditions (left, middle). The number of tubules was counted (right). E. Western blot analysis of HIF-1α, Notch1, Flk1, and p-Flk1 in C6 glioma cells cultured in these two conditions. GAPDH blot serves as loading control. Data are mean ± SD from three independent experiments. NS, no significant difference.

To assess the effects of exogenous EPCs on transdifferentiation of C6 glioma cells into vascular ECs, we examined dissociated tumors by flow cytometry. We found that about 32.3% of total ECs were positive for GFP in EPC group, which was similar to the control group (Fig. 7A). As we showed before, HIF-1α, Notch1, and Flk1 may be important factors for C6 glioma cells transdifferentiation. Thus, we performed Western blot to detect HIF-1α, Notch1, Flk1, and p-Flk1 expressions in tumors. As Fig. 7B showed, no significant differences were found about the expressions of all these factors in groups with or without EPCs transplantation.

Next, transdifferentiation-induced C6 glioma cells were treated with EPCs-CM or vehicle control for 24 h *in vitro*. Followed by, we detected the transdifferentiation frequency by flow cytometry and performed tube formation assay. Compared with the control, no significant differences were found about the transdifferentiation frequency (Fig. 7C) and the ability of tube formation (Fig. 7D) in EPCs-CM group. In addition, Western blot analysis showed no significant differences about the expressions of HIF-1α, Notch1, Flk1, and p-Flk1 in groups with or without EPCs-CM treatment (Fig. 7E). Taken together, these results suggest that exogenous EPCs exert no promoting effect on C6 glioma cells transdifferentiation.

## Discussion

At least five mechanisms by which glioma achieve neovascularization have been described as follows, vascular co-option [29, 30], angiogenesis [31, 32], vasculogenesis [33, 34], vascular mimicry [7, 35], and GSCs-ECs transdifferentiation [8-10]. Recent studies have demonstrated that GSCs transdifferentiating into vascular ECs possibly contributes to tumor resistance to anti-VEGF therapy currently in use [8 - 10]. Here, we have revealed a new mosaic pattern that exogenous EPCs integrated into the vessels containing the tumor-derived ECs in C6 glioma rat model. Importantly, EPCs, homing to gliomas, exerted no promoting effect on the transdifferentiation of C6 glioma cells into vascular ECs. Therefore, our findings suggest that EPCs may be a best vehicle to deliver the therapeutic genes, targeting GSCs transdifferentiation more sufficiently and effectively.

The concept of existence of cancer stem cells in solid tumors including glioma has revolutionized understanding of tumor biology and gives a plausible explanation for treatment failure [36]. Recent studies provide evidence to show that a population of GSCs within glioma could transdifferentiate into vascular ECs in clinical samples, human GBM xenografts, and mouse GBM model [8 -10]. In our study, to investigate the angiogenesis in glioma, we used the in situ C6 glioma rat model. The C6 glioma cell was chosen because the tumor is hypervascular and easily reproducible [16], moreover, this cell line is mainly composed of cancer stem cells [37]. In our present study, tumor-derived ECs were found in gliomas, in addition, about 31.2% of total ECs were transdifferentiated from C6 glioma cells. To confirm the presence of tumor-derived ECs, C6 glioma cells were induced to transdifferentiate into ECs *in vitro* and showed a strong ability to form vascular networks. All of these suggest that C6 glioma cells could transdifferentiate into vascular ECs. As is reported, GSCs transdifferentiation may contribute to resistance to antiangiogenic therapies in glioma [8 - 10]. Therefore, identifying new therapeutic strategies to target GSCs transdifferentiation in glioma is a high priority.

Stem cells are considered to be used as a delivery vehicle for therapeutic genes to tumors, especially for glioma, because of their migration to pathological lesions [11 - 13]. EPCs residing in the bone marrow can be recruited to the tumor in response to tumor-derived cytokines, where they contribute to vascular development by incorporating into the walls of nascent capillaries [33]. Accumulating studies demonstrate that exogenous EPCs also show active migration and integration into neovasculature of glioma *in vivo* [16, 38 - 40]. However, whether exogenous EPCs integrate into the vessels containing tumor-derived ECs or not remains unknown. Here, we found a new mosaic pattern that exogenous EPCs integrated into the vessels containing tumor-derived ECs in glioma tissues. This new mosaic pattern is different from the traditional “mosaic tumor vessel” that is the lumen formed with both ECs and tumor cells lacking EC markers [41]. It is not previously recognized that EPCs integrate into the vessels containing tumor-derived ECs, which highlight another advantage of exogenous EPCs as a vehicle. Our findings suggest that EPCs may be a best vehicle to deliver the therapeutic genes, targeting GSCs transdifferentiation more sufficiently and effectively.

Our previous studies indicate that magnetic nanoparticles could not impact the biology properties of EPCs after labeling [16, 39] and these labeled EPCs exert no significant pro-growth effects on glioma [38]. However, as we mentioned before, EPCs possibly act as an intermediate in GSCs transdifferentiation into ECs, i.e. GSCs at early stage differentiate to EPCs, and then EPCs differentiate to ECs [9]. Therefore, exogenous EPCs, homing to glioma, may exert some effects on GSCs transdifferentiation. To test our hypothesis, we evaluated the transdifferentiation frequency of C6 glioma cells in gliomas with or without EPCs transplantation. Interestingly, no significant difference was found between these two groups. Additionally, similar results were observed in transdifferentiation frequency and tube formation assay of transdifferentiation-induced C6 glioma cells treated with EPCs-CM or vehicle control *in vitro*. Emerging evidences indicate that HIF-1α [10], Notch [9, 21] and VEGFR2 [9, 22] signaling may mediate distinct steps in the transdifferentiation of GSCs into ECs. Consistent with these reports, our studies confirmed the role of HIF-1α, Notch1, and Flk1 in C6 glioma cells transdifferentiation. Furthermore, we evaluated the effect of EPCs on HIF-1α, Notch1, Flk1, and p-Flk1 expressions in tumors and cultured C6 glioma cells via EPCs transplantation or EPCs-CM treatment, respectively. Interestingly, no significant difference was found between the EPC/EPCs-CM group and the control. Taken together, all of these findings suggest that exogenous EPCs exert no promoting effect on the transdifferentiation of C6 glioma cells into vascular ECs. Therefore, it is safe to use EPCs as a vehicle delivering the therapeutic genes to target GSCs transdifferentiation.

Additionally, the development of EPCs-based therapies requires accompanying, noninvasive imaging protocol for *in vivo* tracking of transplanted cells. MRI can be used both to non-invasively follow dynamic spatio-temporal patterns of the EPCs targeting allowing for the optimization of treatment strategies and to assess efficacy of the therapy [42]. Iron-labeled cells allow their presence to be visualized and tracked by MRI [40, 43, 44]. In this study, the temporal and spatial migration of intravenously injected, magnetically labeled EPCs was tracked by MRI *in vivo*, which was consistent with the findings by Prussian blue staining. In addition, SWI on high field small animal MR scanner was used to track the incorporation of these EPCs into vessels in glioma. Furthermore, we found a significant correlation between the number of magnetically labeled EPCs estimated from T_2_ maps (△R2 value) correlated well with direct measurements of labeled cell counts by flow cytometry. Therefore, magnetically labeled EPCs could be used as an imaging probe to be tracked and quantified *in vivo* using MRI.

There are several limitations to this study. One potential criticism is that our study did not exclude the possibility that some of GFP^+^ ECs result from exosome-mediated intercellular transfer, although we confirmed the presence of tumor-derived ECs *in vitro*. Recent reports indicate that some mRNA, miRNA, and signaling proteins from cancer cells can be transferred to other cells in tumor microenviroment by extracellular vesicles, including exosomes and microvesicles [45-47]. GFP from C6 glioma cells may be transferred to ECs lining. Therefore, presence of EC antigen and GFP may not mean that all these cells are transdifferentiated from tumor cells. However, emerging evidence unveils that GFP^+^ ECs are all transformed from glioma cells by using transgenic nude mice [10], which can give a support to what we found.

In conclusion, we found a new mosaic pattern that exogenous EPCs integrated into the vessels containing the tumor-derived ECs in C6 glioma rat model. Importantly, these homing EPCs exerted no promoting effect on the transdifferentiation of C6 glioma cells into vascular ECs. Additionally, the temporal and spatial migration of EPCs could be tracked and quantified *in vivo* using MRI. Our findings may provide a rational base for the future application of EPCs as a therapeutic and imaging probe to overcome antiangiogenic resistance for glioma and monitor the efficacy of this treatment.

## Material and Methods

### Cell culture

C6 glioma cells were obtained from cell bank of Chinese Academy of Sciences (Shanghai, China). C6 glioma cells transduced by pGeenPuro virus were done as described [48]. Cells were maintained at 37℃ and 5% carbon dioxide in DMEM/F_12_ (Gibco, Carlsbad, CA) supplemented with 10% fetal bovine serum and 100 units/ml penicillin (Hyclone, Logan, AR). In the transdifferentiation-induction assay, cells were cultured in endothelial differentiation medium, i.e. DMEM/F_12_ supplemented with 10% FBS, 10ng/ml VEGF, 20ng/ml EGF, 10ng/ml bFGF, 10ng/ml IGF-Ⅰ (Sigma-Aldrich, St. Louis, MO), and 1/100 N_2_ supplement (Gibco, Carlsbad, CA), in hypoxia (i.e. 2% O_2_, 5% CO_2_, and 37℃).

Spleen-derived EPCs were obtained as previously described [38] by isolating mononuclear cells using Ficoll density-gradient centrifugation from healthy Sprague-Dawley rats (obtained from the Experimental Animal Center of Daping Hospital, Chongqing, China). After resuspension in DMEM, containing 20% fetal bovine serum, 10^6^ mononuclear cells/cm^2^ were plated on culture flasks. After 3 days of culture, nonadherent cells were discarded by washing with DPBS. When EPCs were at the subconfluent level, they were identified via uptake DiI-labeled acetylated low-density lipoprotein (acLDL) and binding of FITC-labeled lectin-1 (Sigma-Aldrich, St. Louis, MO). To determine cell surface markers, cells were fixed with 4% paraformaldehyde, incubated with antibodies CD34 (Abnova, Taiwan, China) and vWF (Millipore, Bedford, MA). Cells were visualized by laser scanning confocal microscopy (Leica, Heerbrugg, Switzerland) [39].

### Generation of EPCs conditioned medium

EPCs conditioned medium (EPCs-CM) was obtained as previously described [38]. Briefly, EPCs were maintained in DMEM until they were 60% to 70% confluent. The medium in the flask was changed and centrifuged at 800g for 8 minutes to obtain the superntant. The cells were maintained in new DMEM for 3 to 4 days, and the process was repeated and the supernatant collected.

### Cell treatment

C6 glioma cells were treated in various conditions including endothelial differentiation medium in hypoxia, endothelial differentiation medium containing 5 μM of the γ-secretase inhibitor DAPT (Sigma-Aldrich, St. Louis, MO) or 5 μM of the VEGFR2 inhibitor sunitinib (Santa Cruz Biotechnology Inc., Santa Cruz, CA) or 50% (v/v) EPCs conditioned medium (EPCs-CM) in hypoxia, endothelial differentiation medium in normoxia, and DMEM/F_12_ in normoxia. Then, cells were incubated for 24 h.

### *In vitro* angiogenesis assay

EPCs and C6 glioma cells (1×10^4^ cells/well) cultured in various conditions were seeded on a 96-well plate that was precoated with Matrigel (BD Biosciences, San Jose, CA). After incubation for 24 h, the formation of tubule-like structures was examined and counted using phase contrast microscopy (Leica, Heerbrugg, Switzerland). The mean number (±SD) of tubules was assessed by counting five random low power (100×) fields per well, and each experiment was replicated three times.

### EPCs labeling

EPCs were labeled with USPIO (P7228; Guerbet Asia Pacific, Hong Kong, China) as described previously [38]. After USPIO labeling, some of these cells were labeled with fluorescent dye DiI (Invitrogen, Grand Island, NY), others were labeled with CellTracker™ Green CMFDA (Invitrogen, Grand Island, NY) according to the manufacturer's protocol, washed, resuspended at 1×10^6^ cells per ml. Cells labeled with CMFDA were prepared for transplantation to the glioma-bearing rats which were used for flow cytometry.

### Animal model

The use of laboratory animals was in compliance with the guideline of National Institute of Health. All animal experiments were performed according to a protocol approved by the Animal Use Subcommittee. Forty eight healthy adult male Sprague-Dawley rats weighting 150-200 g were anesthetized and placed in a stereotaxic frame (RWD Life Science,Shenzhen, China). A burr hole was drilled in the skull 1 mm anterior to the bregma and 3 mm lateral to the midline. The needle of microsyringe advanced to the depth of 5 mm and 1×10^6^ C6 glioma cells resuspended in 10μL DPBS were injected to establish the in situ brain glioma model.

Animals were randomized to the EPC group (24 rats) and control group (24 rats). Rats in the EPC group were transplanted with double labeled EPCs (1×10^6^) suspended in 1ml DPBS via tail vein on 10 days after the in situ glioma established. Rats in the control group were administrated equivalent volume of DPBS.

### *In vivo* magnetic resonance imaging

Magnetic resonance imaging (MRI) was performed with a Bruker BioSpec 7 T/20 cm system (Bruker, Ettlingen, Germany) using a head surface coil and a 3 T MR system (Magnetom Verio, Siemens Medical Solutions, Erlangen, Germany) using a specific small animal head coil (Shanghai Chenguang Medical Technologies Co. LTD, Shanghai, China) before EPCs transplantation and on 1, 3, 5, 7 days post-transplantation of EPCs.

To track the migration of the USPIO-EPCs in glioma, 3.0 T MR system was performed. The MRI sequences included a turbo spin-echo (TSE) T_2_-weighted sequence (repetition time = 4210 ms; echo time = 73 ms; field of view = 7×7 cm^2^; slice thickness = 1.5 mm; distance factor = 5%), T_2_ maps sequence (repetition time = 1500 ms; echo time = 15, 30, 45, 60 ms; field of view = 7×7 cm^2^; slice thickness = 2.0 mm; distance factor = 20%).

For further study of the USPIO-EPCs integrating into the vessels in glioma, 7.0 T MR system was performed. The MRI sequences included a TSE T_2_-weighted sequence (repetition time = 2500 ms; echo time = 45 ms; field of view = 3.5×3.5 cm^2^; slice thickness = 0.5 mm; RARE factor = 8; NEX=4), a flash 3d-T2-susceptibility weighted imaging (SWI) sequence (repetition time = 700 ms; echo time = 18 ms; field of view = 3.5×3.5 cm^2^; slice thickness =0.5 mm; flip angle = 40.0°; NEX=8) All data were delivered to the post-processing workstation to perform imaging processing and analysis.

### Prussian blue staining

After in vivo MRI, rats were sacrificed by overdose anesthesia and perfused with saline and 4% paraformaldehyde through the left ventricle to drain blood. Tumors and surrounding tissues were divided in half for frozen sections, and paraformaldehyde fixation with embedding in paraffin. 3μm sections were cut from tumors embedded in paraffin, and then stained with Prussian blue to determine the distribution of USPIO-EPCs in gliomas.

### Immunofluorescent staining of cytospins and tumor sections

For cytospins, C6 glioma cells in endothelial differentiation medium were seeded on coverslips and cultured for 24 h in hypoxia. Tumors were collected and then divided for frozen section at 5μm thickness. Sections were fixed in 4% paraformaldehyde and blocked with 10% goat serum. The primary antibodies used in this study were as follows: rabbit anti-von Willebrand factor (vWF) (Millipore, Bedford, MA), rabbit anti-CD34, mouse anti-CD31, mouse anti-Flk1 (Santa Cruz Biotechnology Inc., Santa Cruz, CA), and goat anti-Notch1 (Santa Cruz Biotechnology Inc., Santa Cruz, CA). The secondary antibodies used were as follows (all from Invitrogen, Grand Island, NY): Alexa Fluor 555-donkey anti-rabbit IgG, Alexa Fluor 568-rabbit anti-mouse IgG, Alexa Fluor 568-rabbit anti-goat IgG, Alexa Fluor 647-rabbit anti-mouse IgG, Alexa Fluor 647-donkey anti-rabbit IgG. Antibodies were diluted in antibody diluent (Beyotime, Jiangsu, China), and incubations were done at room temperature. The images were captured by confocal laser scanning microscopy (Leica, Heerbrugg, Switzerland), and the obtained images were processed by Adobe Photoshop 7.0 (Adobe System Inc., San Jose, CA).

### Transmission electron microscopy analysis

The brain was dissected out and the tumor tissue was randomly selected and divided into some pieces of about 1mm^3^, which were fixed with 2.5% glutaraldehyde for several days at 4℃. After that, according to the standard procedures, semi-thin and ultra-thin sections were made and stained with uranyl acetate and lead citrate, and then viewed using an transmission electron microscope (Philips Tecnai-10, Netherlands).

### Flow cytometry

The brain tumors were dissociated using a Neural Tissue Dissociation Kit (Miltenyi Biotec, Bergisch Gladbach, Germany), and C6 glioma cells were collected after transdifferentiation induction. These cells were stained with the following antibodies according to the manufacturer's protocol: phycoerythrin (PE) anti-CD31 (BD Biosciences, San Jose, CA), anti-Fluorescein/Oregon Green mouse IgG (Invitrogen, Grand Island, NY), and Alexa Fluor 647-rabbit anti-mouse IgG (Invitrogen, Grand Island, NY). They were then analyzed on a BD LSR I flow cytometer (BD Biosciences, San Jose, CA).

### Western blotting

Protein extraction and Western blotting were done as described [48]. Briefly, proteins from C6 glioma cells cultured in various conditions and glioma tissues were extracted and subjected to SDS/PAGE. Proteins were transferred to polyvinylidene difluoride membranes and probed with antibodies for HIF-1α, Notch1, Flk1, p-Flk1 and GAPDH (all from Santa Cruz Biotechnology Inc., Santa Cruz, CA), respectively. After incubation with secondary antibodies, the proteins were detected by enhanced chemiluminescence and quantified using a Gel Doc 2000 Imager (Bio-Rad, Hercules, CA). Each experiment was replicated three times.

### Statistical analysis

Data are presented as mean ± standard deviation (SD). Statistical analysis included *t* test for two groups, one-way ANOVA or two-way ANOVA for multiple groups. Linear regression analysis was performed to compare the correlation between number of labeled EPCs detected by flow cytometry and corresponding MRI △R2 values. Statistical analysis was performed using SPSS software, version 18.0 (SPSS Inc., Chicago, IL). Significance was defined as p<0.05.
